# General practitioners’ opinions of generative artificial intelligence in the UK: An online survey

**DOI:** 10.1177/20552076251360863

**Published:** 2025-07-17

**Authors:** Anna Kharko, Carolina Garcia Sanchez, Josefin Hagström, Jens Gaab, Cosima Locher, Brian McMillan, David Sundemo, Charlotte Blease

**Affiliations:** 1 Participatory eHealth and Health Data Research Group, Department of Women's and Children's Health, 8097 Uppsala University, Uppsala, Sweden; 2 Centre for Primary Care and Health Services Research, 5292 University of Manchester, Manchester, UK; 3 Clinical Psychology and Psychotherapy, Faculty of Psychology, 27209 University of Basel, Basel, Switzerland; 4 Clinical Psychology and Psychosomatics, Faculty of Psychology, 27209 University of Basel, Basel, Switzerland; 5 General Practice/Family Medicine, School of Public Health and Community Medicine, Institute of Medicine, 70712 Sahlgrenska Academy, University of Gothenburg, Gothenburg, Sweden; 6 Digital Psychiatry, Beth Israel Deaconess Medical Center, 1811 Harvard Medical School, Boston, USA

**Keywords:** Artificial intelligence, AI, generative AI, large language models, LLM, general practice, general practitioner, GP, doctor, primary care, education, training, survey opinions, survey research

## Abstract

**Background:**

The rapid rise of large language model-based generative artificial intelligence (AI) tools, such as chatbots and AI scribes, has prompted interest in their clinical applicability. This study examined the perspectives of *general practitioners* (GPs) in the UK regarding the impact of generative AI on clinical practice.

**Methods:**

In January 2025, a national online survey of 1005 GPs, stratified by region, was administered via the UK's largest clinical marketing platform. The survey gathered data on attitudes towards generative AI, its perceived effects on clinical tasks, care delivery and respondent demographics. Group differences and correlations between opinions and respondent characteristics were analysed.

**Results:**

Among the 1005 respondents, 50% were male, 54% aged 46 or older, and 83% practised in England. Only 11% were encouraged by their employer to use generative AI tools, and 5% had received training. Most agreed that AI could improve documentation (69%) and patient information gathering (57%), and 59% expected increased efficiency in care. A similar proportion (59%) anticipated that patients may rely on AI instead of seeking medical care. The majority (79%) agreed that training is needed, while 71% disagreed that AI could enhance empathic communication. Male GPs were more likely to view AI as beneficial across tasks, while female GPs more often emphasised training needs. Older GPs expressed concern about potential inequities stemming from AI use.

**Conclusions:**

While GPs were cautiously optimistic about generative AI – particularly for documentation and data collection – scepticism persisted. In particular, around empathy and equity, highlighting the need for guidance on AI integration into primary care.

## Background

Since the release of ChatGPT in late 2022, interest in *large language model* (LLM)-powered tools has surged.^1^ Underpinned by generative *artificial intelligence* (AI), these tools create novel content thanks to prior training on large datasets.^2–4^ In healthcare, the integration of generative AI is gradually increasing.^5^ General-purpose chatbots, such as ChatGPT,^1^ and domain-specific models pre-trained on medical data, like Med-PaLM,^6^ are entering clinical settings alongside ‘ambient’ AI scribes, such as Nuance DAX,^7^ which generate clinical documentation from consultation audio recordings.^8,9^ These systems aim to support clinicians with documentation tasks and improve accessibility for patients.^10,11^

Despite their potential to improve healthcare workflows, generative AI tools still face significant limitations.^12,13^ Under current UK regulation, generative AI intended for medical purposes requires medical device certification, thus limiting its use in clinical settings.^14^ Other limitations include producing inaccurate information, known as ‘hallucinations’,^15,16^ and perpetuating biases, raising concerns over patient safety and privacy.^15,17–19^ Practical challenges persist, notably the absence of clear guidance on how clinicians should incorporate generative AI into their workflows.^20^ Nonetheless, growing adoption has spurred research into their accuracy and efficiency.

A growing body of research suggests that in experimental settings, generative AI can improve clinical documentation.^9,12,21–26^ Few studies, however, have consulted healthcare professionals, especially frontline clinicians, on their use and opinions about AI in clinical practice. Initial survey research suggests clinicians are interested in generative AI to reduce administrative burdens, which are known drivers of burnout.^27–29^ A 2024 report by the Alan Turing Institute, UK, found, contingent on clinical speciality, that as many as every fifth polled UK doctor had experimented with generative AI tools and a majority expected a positive impact on productivity.^30^ US data echo the trend of rapid adoption, with a large proportion of physicians viewing AI as potentially valuable for reducing documentation time and alleviating burnout.^31,32^ Early peer-reviewed studies, such as one mixed-methods survey of 47 primary care doctors, found generally positive sentiments towards AI, though questions were raised about meaningful workflow integrations.^33^ Another small US survey of 138 psychiatrists found that every fourth respondent had already used generative AI tools, with most agreeing AI will benefit documentation practices.^34^ Still, robust, peer-reviewed large-scale evidence remains lacking – not only on how doctors use these tools, but also on their views of its potential.

We conducted a study exploring how UK *general practitioners* (GPs) are incorporating generative AI tools into everyday clinical workflows. Building on our previous survey,^35^ we examined how attitudes have shifted 1 year later, and to what extent GPs are adopting these tools for clinical tasks.

## Methods

We used the 2025 data from the annual survey of UK GPs’ opinions on AI in clinical practice, the GPAI-UK-2025 Survey.^35^ Since its launch in 2024, the GPA-UK Survey polls a random sample of a thousand verified GPs on their use and opinions of generative AI in clinical practices. The study is reported following the *Checklist for Reporting Results of Internet E-Surveys* (CHERRIES); see Appendix 1.

### Survey design

The GPAI-UK-2025 Survey comprised four sections with 17 closed-ended and one open-ended question (see Appendix 2). Compared to the 2024 version, the questions remained the same, with minor changes to the wording of some answer options to improve clarity. In *Section A*, participants were asked whether they had used generative AI in clinical practice. Those who responded affirmatively were prompted to indicate which AI tools, for which clinical tasks, and whether they perceived them to reduce workload. Further questions explored whether, within the past year, their employer had encouraged or prohibited the use of generative AI and whether they had received any related professional training, without specifying the content, on its use. Results of this section are reported elsewhere.^36^ *Section B* focused on GPs’ opinions about generative AI's impact on various aspects of clinical practice. The selection of questions and answers in this section was guided by our earlier survey and structured around key clinical functions in general practice.^35,37^ The current study reports on these findings. *Section C* included an optional open-ended question allowing participants to provide general comments. Given the depth of these responses, a separate qualitative analysis was conducted, which is reported in another article^35^ to allow for full exploration of emergent themes. Finally, *Section D* collected demographics, such as age, gender, role, place, and size of practice. Most questions were single-choice. All closed-ended questions were mandatory. The final version was pre-tested with five UK GPs and designed for completion within five minutes.

### Survey administration

*As in 2024*, the survey was conducted among GPs registered with Doctors.net.uk, the largest professional network for UK doctors registered with the *General Medical Council* (GMC). At the time of data collection, the network had 254,741 members, accounting for approximately 67% of the 390,000 registered doctors in the UK. The survey was distributed as part of a monthly ‘omnibus survey’ by Doctors.net.uk that polls on various topics related to medicine and healthcare. Our goal was to achieve 1000 complete responses, which required answering all closed-ended questions. Sampling was stratified by regions, based on data from the GMC Data Explorer. It was not possible to include or exclude GPs who had previously completed the 2024 survey, as both the previous and the current versions were anonymous. Invitations were sent via email or displayed as homepage advertisements on Doctors.net.uk, depending on participants’ preferred notification settings. Respondents were compensated with a shopping voucher valued at GB£7.50 (US$ 8.80 or EUR€8.83). The survey remained open 7–26 January 2025 and closed after reaching 1005 responses. As the survey was conducted as part of a scheduled monthly omnibus administered by Doctors.net.uk, and a denominator is not available, a conventional response rate cannot be calculated. However, during the survey period, approximately 25,500 GPs were active on the platform, providing context for the scale of the sampling frame.

### Data analysis

The present study focuses on findings related to GPs’ opinions about generative AI tools in clinical practice (*Section B*). These results are analysed and interpreted in conjunction with data on generative AI use (*Section A*) and respondent characteristics (*Section D*). Descriptive statistics were employed to summarise demographics, experiences, and opinions about generative AI in clinical practice. The opinion survey items used a 7-point Likert scale agreement levels, ranging from 1 ‘*Strongly disagree*’ to 6 ‘*Strongly agree*’ with 7 ‘*Don’t know*’. Median agreement ratings were calculated excluding ‘*Don’t know*’ responses. To examine associations between variables, Spearman's rho was used for correlation analyses, while Mann–Whitney *U* test was applied for group difference analyses. These non-parametric methods were chosen due to their suitability for ordinal data. ‘*Don’t know*’ responses were excluded for both. To illustrate absolute group differences, Likert scale responses were dichotomised (1–3 = ‘*Disagree*’, 4–6 = ‘*Agree*’), allowing the calculation of percentage agreement across subgroups. Two-tailed significance level was pre-set to 0.05. Analyses were carried out by JH (IBM SPSS Statistics, v24) and AK (JASP, v0.18.3) and independently verified by CGS (‘pandas’ library v2.2.3 in Python v3.11.9). Figures were created by AK using Datawrapper and Microsoft Office Excel 2019.

### Ethical considerations

Informed consent was obtained before respondents proceeded with the survey. Data collection took place through the Doctors.net.uk platform, which guarantees encryption and full anonymisation, preventing any linkage between responses and identifiable information. Personal identifiers, such as email addresses, were removed before the dataset was transferred to the research team. The platform complies with the European Union's General Data Protection Regulation. Ethical approval for the study was granted by the Faculty of Psychology, University of Basel (#030-24-1).

## Results

### Respondent characteristics

Among the 1005 participants, half identified as men (50%), and half were aged 46 years and older (54%); see Table 1. Forty-four percent of GPs worked as GP Partners or Principals (i.e. were part-owners of the GP practice), and half were based in a clinical practice with over 10,000 patients (52%).

**Table 1.** Respondent characteristics.

	Count	%
**Gender**		
Woman	486	48%
Man	506	50%
Preferred not to say	13	1
**Age**		
35 years or younger	86	9%
36–45 years	373	37%
46–55 years	366	36%
56 years or older	180	18%
**Role**		
GP Partner or Principal	439	44%
Salaried GPs	380	38%
Locum GPs	150	15%
GP Registrar	36	4%
**GP practice size**		
Up to 5000 patients	112	11%
5001–7500 patients	165	16%
7501–10,000 patients	202	20%
10,001–12,500 patients	170	17%
12,501 patients or more	356	35%

**Figure 1.**
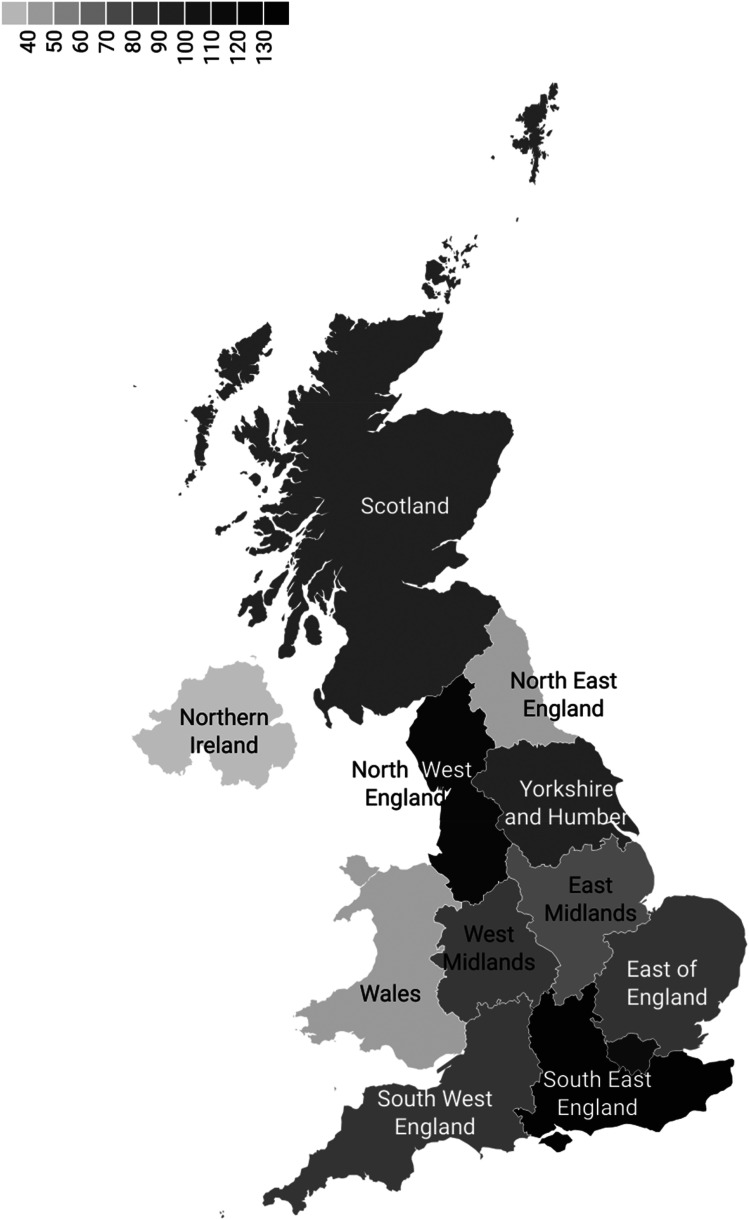
Geographical distribution of participating GPs by NHS region.

Most responses came from England (*n* = 831, 81%), where most were located in the Midlands (*n* = 154, 15%); see Figure 1. Our sample was comparable to the GMC Register in regional distribution but differed in gender (see Appendix 3). Based on GMC Data Explorer, 58% women GPs were registered at the beginning of 2025 compared to 48% of women in our sample.

### Generative AI in the workplace

As reported previously, 11% (*n* = 107) were encouraged by their employer to use generative AI tools in the workplace, 5% (*n* = 52) received training for it, and 3.5% (*n* = 35) were prohibited from using it (see Appendix 3). Seventeen percent (*n* = 176) reported that the use of generative AI had reduced their workload.

### GPs’ opinions about generative AI

**Figure 2.**
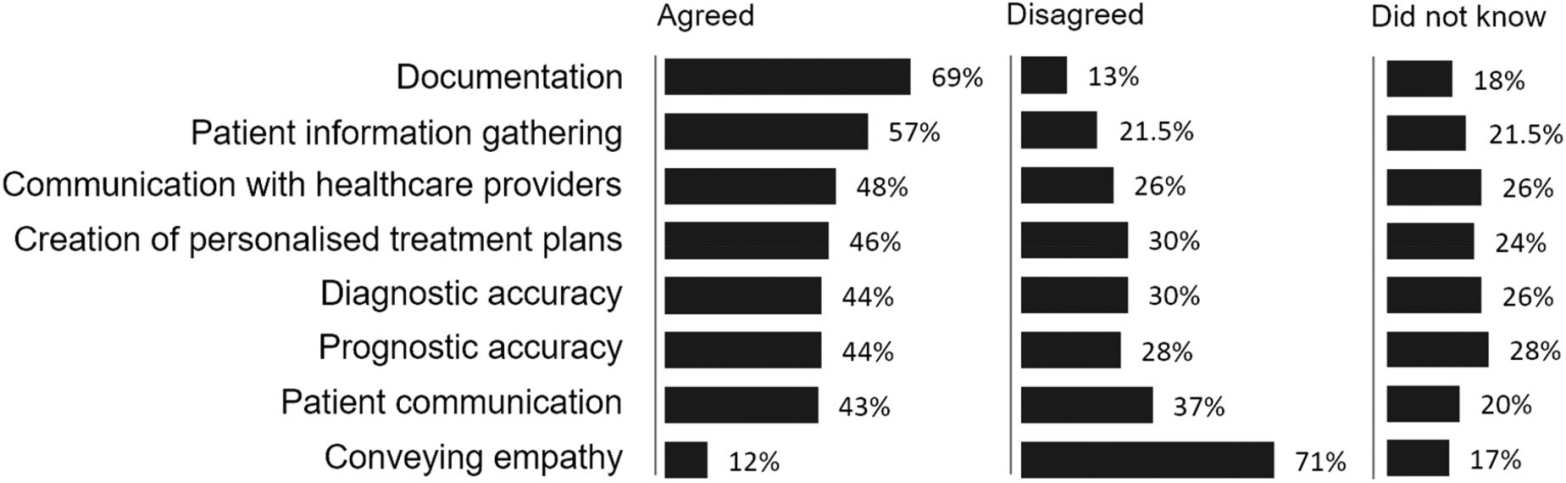
Opinions about how generative AI tools will improve various aspects of GP work.

When considering how generative AI will affect various aspects of their work, respondents most agreed it would improve: documentation (*n* = 694, 69%), patient information gathering (*n* = 573, 57%), and communication with other healthcare providers (*n* = 483, 48%); see Figure 2. GPs most disagreed that AI could improve conveying empathy (*n* = 712, 71%) and were most unsure about it improving prognostic accuracy (*n* = 276, 28%).

Predicting how the use of generative AI will impact healthcare, respondents most agreed that: they will need more support or training in understanding such tools (*n* = 798, 79%), AI use will increase efficiencies in healthcare (*n* = 591, 59%), and more patients will rely on AI tools instead of seeking medical attention (*n* = 589, 59%). GPs most disagreed that patient privacy will increase (*n* = 605, 60%); see Appendix 3.

### Associations between GPs’ opinions and characteristics

Analysing opinions on the use of generative AI tools in clinical practice, significant differences were observed for several characteristics.

#### Gender

Men agreed to a greater extent that AI will improve diagnostic accuracy (59% of men compared to 41% of women; *U* =  57,893.0, *p* < 0.001), patient communication (59% compared to 48%; *U* =  67,049.0, *p* < 0.001), conveying empathy (17% compared to 12%; *U* =  74,680.5, *p* = 0.002), creation of personalised treatment plans (65% compared to 57%; *U* =  63,323.5, *p* = 0.010), and prognostic accuracy (64% compared to 58%; *U* =  59,240.5, *p* = 0.029). More male GPs also believed that generative AI tools will decrease patient harm (51% compared to 39%; *U* =  46,213.5, *p* < 0.001), and increase patient privacy (17% compared to 10%; *U* =  53,130.0, *p* = 0.007). Meanwhile, a greater proportion of women anticipated that GPs will need more support or training in understanding these tools (93% of women compared to 89% of men; *U* =  81,972.5, *p* < 0.001).

#### Age

Respondents’ age was weakly correlated with opinions about generative AI. Older GPs – especially those aged 56 and older – anticipated that these tools will improve diagnostic (ρ = 0.08, *p* = 0.032, *n* = 740, e.g. 71% of GPs ≥56 agreed vs 58% ≤ 35) and prognostic accuracy (ρ = 0.08, *p* = 0.035, *n* = 729, e.g. 69% of GPs ≥56 agreed vs 56% ≤ 35); however, they also perceived that the tools will increase inequities in care delivery (ρ = 0.08, *p* = 0.044, *n* = 681, e.g. 44% of GPs ≥56 agreed vs 61% ≤ 35). Younger GPs agreed that these tools will improve communication with other healthcare providers (ρ = −0.07, *p* = 0.049, *n* = 744, ≥ 54% in all older groups vs 39% in ≤35).

#### Workplace generative AI training

Prior training was significantly associated with greater confidence that generative AI could enhance GPs’ ability to gather patient information (83% of those who received training compared to 72% of those who had not received training; *U* =  13,862.0, *p* = 0.014) and patient communication (69% compared to 53%; *U* =  12,720.0, *p* = 0.006). Trained respondents were also less inclined to agree that GPs need more support and training (84% compared to 91%; *U* =  16,948.5, *p* = 0.039).

## Discussion

### Principal findings

The study explored the opinions of UK GPs on the role of generative AI in clinical practice. Most GPs expected benefits from AI use in a variety of clinical tasks, including documentation, patient information gathering, and overall healthcare efficiency. While this likely reflects familiarity with current AI applications, such as documentation, e.g. summarisation, we also interpret it as recognition of a clear practical benefit. Many believed that patients will rely on AI tools instead of seeking medical consultation, which is notable as GPs overwhelmingly disagreed that AI will be able to improve empathy conveying and believed these tools will pose risks to patient privacy. We recognise that this concept ‘convey empathy’, may have been interpreted in different ways, though our intention was to capture whether AI might support, rather than replace, GPs’ communication to become more empathic.

There was widespread agreement that, as a result of AI integration in healthcare, GPs will need support or training in understanding it (79%). Opinions varied by gender, age, and experience of prior training. Men were more prone to perceive a positive impact of AI, while women were more likely to endorse the need for further support or training.

Notably, only 11% of respondents reported that their employer had encouraged the use of generative AI. This suggests that much of the current use is occurring informally, without institutional oversight – a pattern some have described as ‘undercover use’.^38^ The absence of organisational governance may shape clinicians’ attitudes, especially around trust, uncertainty, and risk perception. Without clear policies, liability frameworks or institutional assurances, doctors may lack the structural support needed to feel confident in adopting these tools more fully.

**Figure 3.**
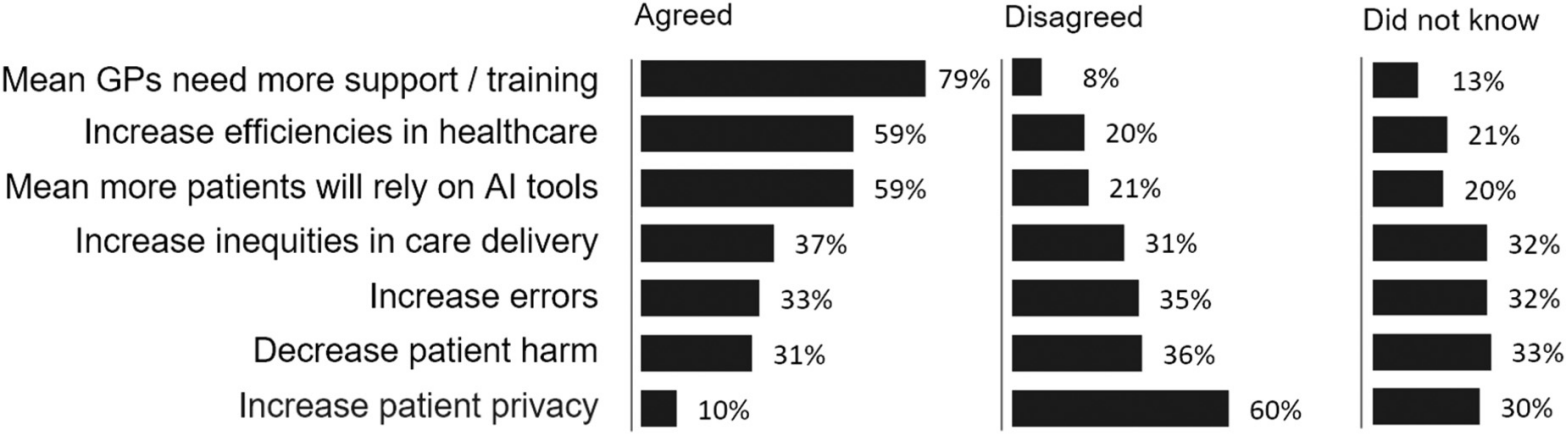
Opinions about how generative AI tools will impact various aspects of healthcare.

Across several opinion items, a substantial proportion of respondents selected ‘*Don’t know*’ – in particular regarding the impact on care inequities (32%), risk of errors (32%), and potential to reduce patient harm (33%). Where respondents did express a view, opinions were polarised, with agreement and disagreement evenly split; see Figure 3. This uncertainty may reflect limited training in generative AI tools. As adoption increases and GPs gain first-hand experience, we can expect a decline in ambiguity and a clearer articulation of both the benefits and limitations of these technologies.

### Comparison with prior work

The present findings align broadly with those of the GPAI-UK-2024 Survey, though some divergences emerged (see Appendix 3). Similar to 2024, GPs agreed that the use of generative AI will increase the need for training (80% in 2024 → 79% in 2025), improve patient information gathering (56% → 57%) and diagnostic accuracy (40% → 44%), and that more patients will rely on AI tools instead of seeking medical advice (62% → 59%). The consistent high support for further training on AI tools is not surprising. As many as 1 in 4 UK GPs are already using AI tools – both commercial and purpose-built – in clinical practice.^30,35^ Although adoption of generative AI is steadily increasing across healthcare, a report by NHS Health Education England identified a skills gap among healthcare professionals due to lack of standardised training pathways.^39^ In this context, the persisting belief that generative AI will improve information gathering is striking, likely reflecting clinicians’ preferences for applying AI to laborious tasks such as data collection and synthesis.^40^

Although the proportion of GPs who believed patients would turn to generative AI tools instead of medical care has slightly decreased, it remains high. Supporting this, studies show that AI adoption is no longer limited to clinicians – patients, too, are increasingly turning to these tools for healthcare guidance.^41^ In Australia, 1 in 10 members of the public have reported asking ChatGPT a health-related question.^42^ The uptake of AI chatbots for health queries is likely to be on the rise in the UK as well, not least because of healthcare access challenges. Patients’ own health research is not novel either, as they have long utilised internet search engines, like Google, to help understand symptoms or even self-diagnose.^43^ Compared to traditional web searches, however, LLM-chatbots may offer faster, tailored advice at a lower readability level,^44,45^ which will likely attract more patients. Still, just as with GPs in our survey, opinions among the public are likely to be divided. For example, a survey of the UK general population found that many are sceptical towards AI in healthcare, with 1 in 6 believing it will make care quality worse.^46^ While commercial AI chatbots are easy to access, they may be more prone to hallucinations or exhibit unwanted bias.^47^

We identified some shifts in GPs’ opinions when comparing the 2024 and 2025 GPAI-UK Surveys. In 2025, more clinicians believed generative AI will improve documentation (59% in 2024 → 69% in 2025) and healthcare efficiencies (47% → 59%). This may be due to the rising public awareness of AI, and also the increasing prevalence of medical-grade AI tools across the NHS, such as Heidi and Hepian Write.^48^ In addition to earlier tools like Hepian Write, 2024–2025 saw the launch and scaling of more sophisticated, clinically oriented AI tools. Ambient scribe technologies became more prevalent in NHS pilot sites, including commercial systems like Nuance DAX and proprietary models embedded within EHR systems. New clinical chatbots, such as OpenEvidence, also entered the scene, offering domain-specific LLMs for summarising evidence and supporting clinical decision-making. These developments may have contributed to shifting GP perceptions, particularly the increased belief in AI's utility for documentation and communication tasks. As these tools mature and integrate further into clinical workflows, we anticipate continued evolution in GP attitudes, especially in relation to real-world usability and trust.

While the reasons are not well understood, in 2025, fewer GPs believed that generative AI will increase the risk of inequity in care delivery (55% → 37%). In both surveys, GPs most disagreed about AI being able to improve the delivery of empathy (65% → 71%). This upward trend is particularly striking given prior research suggesting that AI tools, like ChatGPT, can embed cues of empathy effectively in clinical documentation.^9,11,46,49^

Our study further builds upon other research on clinicians’ perspectives on AI. In 2023, a survey of 138 US psychiatrists posed similar questions about the perceived impact of generative AI on mental healthcare.^34^ There, 9 in 10 believed that further training was needed, compared to the 8 in 10 here. Concurrently, an almost identical proportion of respondents in both studies expected AI to improve documentation (45%) and diagnostic accuracy (44%). The emergence of similar perspectives on AI usefulness despite different specialities and healthcare systems is noteworthy. A small 2024 survey of GMC-registered doctors also found only a small minority of respondents to have had formal training on AI (7%, *n* = 272) although over half had used it (56%, *n* = 272).^50^ Both their findings and ours highlight a clear and pressing need for targeted education and robust support to equip clinicians with the skills and confidence to use AI effectively in practice.

Despite the wide range of positives expected by GPs to emerge from the use of generative AI in clinical practice, only 17% among those who used it reported reduced work burdens. This discrepancy may reflect the wide range of tasks generative AI may be applied to, with some use cases being more amenable to efficiency gains than others. For example, Tai-Seale et al. (2024) found no time savings and noted longer replies and reading time when clinicians used AI to draft patient messages. They did, however, perceive improvements in tone and compassion signalling. These mixed findings highlight that AI's impact on work burdens is nuanced and context dependent. It also is particularly relevant when considering the shifting expectations with the increased implementation of AI tools in clinical practice. In other research, clinicians have been cautious that they may be expected to see more patients as a result of integrating AI into the clinical workflow.^33^ In the UK, the use of ambient AI already appears to have reduced patient waiting times,^51^ which is beneficial for patients but raises questions about new pressers on GPs and their capacity to deliver care.

### Strengths and limitations

This is one of the first studies to examine clinicians’ opinions about generative AI in the workplace. While we achieved a large sample (*n* = 1005), the inability to calculate a formal response rate is a limitation. Nonetheless, the sample was stratified by NHS region and broadly representative in terms of age and geography. We acknowledge the underrepresentation of women in our sample, which may reflect differences in survey engagement or platform use. These limitations are important to consider in interpreting the results. A key strength was the inclusion of GMC-registered doctors, stratified by NHS region, which enhanced the representativeness of the findings. The survey, however, used a convenience sample, limited to members of Doctors.net.uk, which may have biased the findings. We also observed a high proportion of ‘*Don’t know*’ answers, likely due to the GPs’ still limited exposure to generative AI tools. Since the survey did not assess GPs' knowledge of generative AI tools, it is not possible to discern how potential limited technical familiarity has affected respondents’ perspectives. Finally, the survey relied on self-report, and past use of AI could not be verified. Given the lack of comprehensive policy, authorised use of generative AI in clinical practice remains unclear, and some respondents may have chosen not to disclose their use or opinions because of this.

### Future directions

Our findings point to several prominent directions for future research. First, while most GPs agreed that further training is needed, it remains unclear what kind of support they envision. A 2024 interview study commissioned by the GMC provides initial insight, including basic education on AI, specific system training, and advice on ethics and data protection.^52^ Future research should explore the types of content clinicians would consider beneficial, as well as which delivery formats they prefer, to ensure the education is engaging and sustainable. Second, many GPs believed patients will adopt generative AI for healthcare purposes, but little is known about wider expectations or preferences regarding patients' use, and its implications for care delivery and doctor–patient relationship.^33^ Further research is needed to explore GPs’ understanding of their professional responsibilities when using AI tools, as well as how their profession may change. An evaluation of GPs’ technical understanding of AI tools could also help clarify whether certain attitudes, particularly regarding expected benefits and risks, are shaped by informed insight or the lack thereof. Research carried out prior to the widespread availability of LLM-chatbots pointed to mixed predictions about the impact of machine learning on clinical practice.^53–55^ Finally, to obtain more robust evidence of trends in GPs’ opinions, a panel survey could be administered, examining the potential changes in views and experiences of the same cohort of clinicians over time.

## Conclusion

This study represents one of the few large-scale surveys exploring UK GPs’ opinions about generative AI. Using a large sample, we focused on the views of GMC-registered UK GPs on the role of AI in primary care. Despite limited training and institutional support, many GPs anticipated benefits – particularly in documentation and patient information gathering. However, uncertainty and scepticism remain, especially regarding generative AI tools’ potential to enhance empathy, equity and patient safety. As our findings suggest that AI use among GPs has increased since the previous year, the continued absence of formal support is concerning. Clear guidance and targeted training are essential to ensure the safe and effective integration of generative AI into clinical practice.

## Supplemental Material

sj-docx-1-dhj-10.1177_20552076251360863 - Supplemental material for General practitioners’ opinions of generative artificial intelligence in the UK: An online survey Supplemental material, sj-docx-1-dhj-10.1177_20552076251360863 for General practitioners’ opinions of generative artificial intelligence in the UK: An online survey by Anna Kharko, Carolina Garcia Sanchez, Josefin Hagström, Jens Gaab, Cosima Locher, Brian McMillan, David Sundemo and Charlotte Blease in DIGITAL HEALTH

sj-docx-2-dhj-10.1177_20552076251360863 - Supplemental material for General practitioners’ opinions of generative artificial intelligence in the UK: An online survey Supplemental material, sj-docx-2-dhj-10.1177_20552076251360863 for General practitioners’ opinions of generative artificial intelligence in the UK: An online survey by Anna Kharko, Carolina Garcia Sanchez, Josefin Hagström, Jens Gaab, Cosima Locher, Brian McMillan, David Sundemo and Charlotte Blease in DIGITAL HEALTH

sj-docx-3-dhj-10.1177_20552076251360863 - Supplemental material for General practitioners’ opinions of generative artificial intelligence in the UK: An online survey Supplemental material, sj-docx-3-dhj-10.1177_20552076251360863 for General practitioners’ opinions of generative artificial intelligence in the UK: An online survey by Anna Kharko, Carolina Garcia Sanchez, Josefin Hagström, Jens Gaab, Cosima Locher, Brian McMillan, David Sundemo and Charlotte Blease in DIGITAL HEALTH
