# Supplementary material for: General practitioners’ opinions of generative artificial intelligence in the UK: An online survey
Source: Digit Health. 2025 Jul 17;11:20552076251360863. doi: 10.1177/20552076251360863 (PMC12276478; doi:10.1177/20552076251360863)
Supplement: sj-docx-1-dhj-10.1177_20552076251360863 - Supplemental material for General practitioners’ opinions of generative artificial intelligence in the UK: An online survey [file sj-docx-1-dhj-10.1177_20552076251360863.docx]

**Appendix** **1.** Checklist for Reporting Results of Internet E-Surveys (CHERRIES)

| **Item Category** | **Checklist item** | **Described in the manuscript** | **Cited from the manuscript** |
| --- | --- | --- | --- |
| **Design** | Describe survey design | Yes | **Methods** > **Survey design** |
| **IRB (Institutional Review Board) approval and informed consent process** | IRB approval | Yes | **Methods** > **Ethical considerations**  Ethical approval for the study was granted by the Faculty of Psychology, University of Basel (#030-24-1) |
|  | Informed consent | Yes | **Methods** > **Ethical considerations**  Informed consent was obtained before respondents proceeded with the survey. |
|  | Data protection | Yes | **Methods** > **Ethical considerations**  Data collection took place through the Doctors.net.uk platform, which guarantees encryption and full anonymisation, preventing any linkage between responses and identifiable information. Personal identifiers, such as email addresses, were removed before the dataset was transferred to the research team. The platform complies with the European Union's General Data Protection Regulation. |
| **Development and pre-testing** | Development and testing | Yes | **Methods** > **Survey design**  The final version was pre-tested with five UK GPs, and designed for completion within 5 minutes. |
| **Recruitment process and description of the sample having access to the questionnaire** | Open survey versus closed survey | Yes | **Methods** > **Survey administration**  The survey was conducted among GPs registered with Doctors.net.uk, the largest professional network for UK doctors registered with the General Medical Council (GMC). |
|  | Contact mode | Yes | **Methods** > **Survey administration**  Invitations were sent via email or displayed as homepage advertisements on Doctors.net.uk, depending on participants' preferred notification settings. |
|  | Advertising the survey | Yes | **Methods** > **Survey administration**  The survey was distributed as part of a monthly ‘omnibus survey’ by Doctors.net.uk that polls on various topics related to medicine and healthcare…Invitations were sent via email or displayed as homepage advertisements on Doctors.net.uk, depending on participants' preferred notification settings. |
| **Survey administration** | Web/E-mail | Yes | **Methods** > **Survey administration**  Invitations were sent via email or displayed as homepage advertisements on Doctors.net.uk, depending on participants' preferred notification settings. |
|  | Context | Yes | **Methods** > **Survey administration**  The survey was conducted among GPs registered with Doctors.net.uk, the largest professional network for UK doctors registered with the General Medical Council (GMC). At the time of data collection, the platform had 254,741 members, accounting for approximately 67% of the 390,000 registered doctors in the UK. |
|  | Mandatory/voluntary | Yes | **Methods** > **Ethical considerations**  Informed consent was obtained before respondents proceeded with the survey. |
|  | Incentives | Yes | **Methods** > **Survey administration**  Respondents were compensated with a shopping voucher valued at GBP 7.50 (USD 8.80 or EUR 8.83). |
|  | Time/Date | Yes | **Methods** > **Survey administration**  The survey remained open January 7 – 26, 2025 and closed after reaching 1,005 responses. |
|  | Randomization of items or questionnaires | No |  |
|  | Adaptive questioning | No |  |
|  | Number of Items | No |  |
|  | Number of screens (pages) | No |  |
|  | Completeness check | Yes | **Methods** > **Survey administration**  Our goal was to achieve 1,000 complete responses, which required answering all closed-ended questions. |
|  | Review step | No |  |
| **Response rates** | Unique site visitor | No |  |
|  | View rate (Ratio of unique survey visitors/unique site visitors) | No |  |
|  | Participation rate (Ratio of unique visitors who agreed to participate/unique first survey page visitors) | No |  |
|  | Completion rate (Ratio of users who finished the survey/users who agreed to participate) | No |  |
| **Preventing multiple entries from the same individual** | Cookies used | No |  |
|  | IP check | No |  |
|  | Log file analysis | No |  |
|  | Registration | No |  |
| **Analysis** | Handling of incomplete questionnaires | Yes | **Methods** > **Survey administration**  Our goal was to achieve 1,000 complete responses, which required answering all closed-ended questions. |
|  | Questionnaires submitted with an atypical timestamp | No |  |
|  | Statistical correction | No |  |

**Reference:**

Eysenbach G. Improving the quality of Web surveys: the Checklist for Reporting Results of Internet E-Surveys (CHERRIES). J Med Internet Res 2004;6:e34.
