# Supplementary material for: General practitioners’ opinions of generative artificial intelligence in the UK: An online survey
Source: Digit Health. 2025 Jul 17;11:20552076251360863. doi: 10.1177/20552076251360863 (PMC12276478; doi:10.1177/20552076251360863)
Supplement: sj-docx-2-dhj-10.1177_20552076251360863 - Supplemental material for General practitioners’ opinions of generative artificial intelligence in the UK: An online survey [file sj-docx-2-dhj-10.1177_20552076251360863.docx]

# **Appendix 2.** GPAI-UK-2025 Survey

**Survey for UK GPs 2025**

The following questions ask about your experiences using generative AI in clinical practice. Generative AI includes tools such as OpenAI's ChatGPT, Google's Gemini (formerly Bard), Microsoft's Bing AI, Nuance DAX Copilot, and others.

| **SECTION A** |
| --- |

**Q1a. Have you ever purposely used generative AI tools to assist you in any aspect of clinical practice?**

𐩒 Yes (*If answered Yes, answer Q1b-d)*

𐩒 No

**Q1b. What tools have you used?**
*(Please select all that apply)*

☐ ChatGPT

☐ Microsoft Copilot

☐ Bing AI

☐ Bard

☐ Claude

☐ Med-PaLM

☐ Other (please specify): ____________

**Q1c. For what purpose(s) have you used generative AI tools?**
*(Please select all that apply)*

☐ Documentation after patient appointments

☐ Differential diagnosis

☐ Treatment options

☐ Referrals

☐ Patient summarization/timelines from prior documentation

☐ Medical certification e.g., for employment

☐ Other (please specify): ____________

**Q1d. In general, have these tools reduced your work burdens?**

𐩒 Yes

𐩒 No

**Q2a. In the last 12 months, has your employer encouraged you to use generative AI tools in your work?**

𐩒 Yes

𐩒 No

𐩒 Don’t know

**Q2b. In the last 12 months, has your employer prohibited you from using generative AI tools in your work?**

𐩒 Yes

𐩒 No

𐩒 Don’t know

**Q2c. In the last 12 months, have you participated in any professional training provided by your employer on the use of generative AI tools?**

𐩒 Yes

𐩒 No

| **SECTION B** |
| --- |

**Q3. Generative AI tools will improve my work related to…**

|  | Strongly Disagree | Disagree | Somewhat Disagree | Somewhat Agree | Agree | Strongly Agree | Don't Know |
| --- | --- | --- | --- | --- | --- | --- | --- |
| Patient information gathering | 𐩒 | 𐩒 | 𐩒 | 𐩒 | 𐩒 | 𐩒 | 𐩒 |
| Diagnostic accuracy | 𐩒 | 𐩒 | 𐩒 | 𐩒 | 𐩒 | 𐩒 | 𐩒 |
| The creation of personalized treatment plans | 𐩒 | 𐩒 | 𐩒 | 𐩒 | 𐩒 | 𐩒 | 𐩒 |
| Prognostic accuracy | 𐩒 | 𐩒 | 𐩒 | 𐩒 | 𐩒 | 𐩒 | 𐩒 |
| Conveying empathy | 𐩒 | 𐩒 | 𐩒 | 𐩒 | 𐩒 | 𐩒 | 𐩒 |
| Documentation | 𐩒 | 𐩒 | 𐩒 | 𐩒 | 𐩒 | 𐩒 | 𐩒 |
| Patient communication | 𐩒 | 𐩒 | 𐩒 | 𐩒 | 𐩒 | 𐩒 | 𐩒 |
| Communication with other healthcare providers | 𐩒 | 𐩒 | 𐩒 | 𐩒 | 𐩒 | 𐩒 | 𐩒 |

**Q4. To what extent do you agree that use of generative AI tools in healthcare will…**

|  | Strongly Disagree | Disagree | Somewhat Disagree | Somewhat Agree | Agree | Strongly Agree | Don't Know |
| --- | --- | --- | --- | --- | --- | --- | --- |
| Increase errors | 𐩒 | 𐩒 | 𐩒 | 𐩒 | 𐩒 | 𐩒 | 𐩒 |
| Decrease patient harm | 𐩒 | 𐩒 | 𐩒 | 𐩒 | 𐩒 | 𐩒 | 𐩒 |
| Increase patient privacy | 𐩒 | 𐩒 | 𐩒 | 𐩒 | 𐩒 | 𐩒 | 𐩒 |
| Increase inequities in care delivery | 𐩒 | 𐩒 | 𐩒 | 𐩒 | 𐩒 | 𐩒 | 𐩒 |
| Mean more patients will rely on AI tools instead of seeking medical attention | 𐩒 | 𐩒 | 𐩒 | 𐩒 | 𐩒 | 𐩒 | 𐩒 |
| Mean GPs need more support/training in understanding them | 𐩒 | 𐩒 | 𐩒 | 𐩒 | 𐩒 | 𐩒 | 𐩒 |
| Increase efficiencies in healthcare | 𐩒 | 𐩒 | 𐩒 | 𐩒 | 𐩒 | 𐩒 | 𐩒 |

**Q5. As a result of the use of generative AI, the meaningfulness of my role as a GP will…**

𐩒 Increase

𐩒 Not change

𐩒 Decrease

**Q6. Please think about how your practice could be affected by generative AI. In your opinion, these tools will:**

𐩒 Decrease my risk of having legal action taken against me

𐩒 Increase my risk of having legal action taken against me

𐩒 Neither decrease nor increase my risk

𐩒 Don’t know

| **SECTION C** |
| --- |

**Q1. Please add any comments about the topic or the survey.** [Optional]

*Please add 1-2 brief comments.*

|  |
| --- |

| **SECTION D** |
| --- |

**S1. Which of the following best describes your role?**

𐩒 GP Partner/Principal

𐩒 Salaried GP

𐩒 Locum GP

𐩒 GP Registrar

**S2. Where are you currently practicing?**

𐩒 England

𐩒 London

𐩒 South West

𐩒 South East

𐩒 West Midlands

𐩒 East Midlands

𐩒 East of England

𐩒 Yorkshire and Humber

𐩒 North East

𐩒 North West

𐩒 Scotland

𐩒 Wales

𐩒 Northern Ireland

**D1. Are you...**

𐩒 Male

𐩒 Female

𐩒 Prefer not to say

**D2. Are you…**

𐩒I35 or under

𐩒I36 to 45

𐩒I46 to 55

𐩒I56 or older

**D3. Which of the following best describes the area where your practice is based?**

𐩒 NHS North Central London ICB

𐩒 NHS North East London ICB

𐩒 NHS North West London ICB

𐩒 NHS South East London ICB

𐩒 NHS South West London ICB

𐩒 NHS Bath and North East Somerset, Swindon and Wiltshire ICB

𐩒 NHS Bristol, North Somerset and South Gloucestershire ICB

𐩒 NHS Cornwall and the Isles of Scilly ICB

𐩒 NHS Devon ICB

𐩒 NHS Dorset ICB

𐩒 NHS Gloucestershire ICB

𐩒 NHS Somerset ICB

𐩒 NHS Bedfordshire, Luton and Milton Keynes ICB

𐩒 NHS Buckinghamshire, Oxfordshire and Berkshire West ICB

𐩒 NHS Frimley ICB

𐩒 NHS Hampshire and Isle of Wight ICB

𐩒 NHS Kent and Medway ICB

𐩒 NHS Surrey Heartlands ICB

𐩒 NHS Sussex ICB

𐩒 NHS Birmingham and Solihull ICB

𐩒 NHS Black Country ICB

𐩒 NHS Coventry and Warwickshire ICB

𐩒 NHS Herefordshire and Worcestershire ICB

𐩒 NHS Shropshire, Telford and Wrekin ICB

𐩒 NHS Staffordshire and Stoke-on-Trent ICB

𐩒 NHS Derby and Derbyshire ICB

𐩒 NHS Leicester, Leicestershire and Rutland ICB

𐩒 NHS Lincolnshire ICB

𐩒 NHS Northamptonshire ICB

𐩒 NHS Nottingham and Nottinghamshire ICB

𐩒 NHS Cambridgeshire and Peterborough ICB

𐩒 NHS Hertfordshire and West Essex ICB

𐩒 NHS Mid and South Essex ICB

𐩒 NHS Norfolk and Waveney ICB

𐩒 NHS Suffolk and North East Essex ICB

𐩒 NHS South Yorkshire ICB

𐩒 NHS Humber and North Yorkshire ICB

𐩒 NHS West Yorkshire ICB

𐩒 NHS North East and North Cumbria ICB

𐩒 NHS Cheshire and Merseyside ICB

𐩒 NHS Greater Manchester ICB

𐩒 NHS Lancashire and South Cumbria ICB

𐩒 NHS Ayrshire and Arran

𐩒 NHS Borders

𐩒 NHS Dumfries and Galloway

𐩒 NHS Fife

𐩒 NHS Forth Valley

𐩒 NHS Grampian

𐩒 NHS Greater Glasgow and Clyde

𐩒 NHS Highland

𐩒 NHS Lanarkshire

𐩒 NHS Lothian

𐩒 NHS Shetland

𐩒 NHS Tayside

𐩒 NHS Western Isles (Bòrd SSN nan Eilean Siar)

𐩒 Aneurin Bevan University Health Board

𐩒 Betsi Cadwaladr University Health Board

𐩒 Cardiff and Vale University Health Board

𐩒 Cwm Taf Morgannwg University Local Health Board

𐩒 Hywel Dda University Health Board

𐩒 Powys Teaching Health Board

𐩒 Swansea Bay University Local Health Board

𐩒 Belfast Health and Social Care Trust

𐩒 Northern Health and Social Care Trust

𐩒 South Eastern Health and Social Care Trust

𐩒 Southern Health and Social Care Trust

𐩒 Western Health and Social Care Trust

𐩒 Other

**D4. How many patients do you have on your practice list?**

𐩒IUp to 5,000 patients

𐩒I5,001-7,500 patients

𐩒I7,501-10,000 patients

𐩒I10,001-12,500 patients

𐩒I12,501 patients or more
