# Supplementary material for: General practitioners’ opinions of generative artificial intelligence in the UK: An online survey
Source: Digit Health. 2025 Jul 17;11:20552076251360863. doi: 10.1177/20552076251360863 (PMC12276478; doi:10.1177/20552076251360863)
Supplement: sj-docx-3-dhj-10.1177_20552076251360863 - Supplemental material for General practitioners’ opinions of generative artificial intelligence in the UK: An online survey [file sj-docx-3-dhj-10.1177_20552076251360863.docx]

**Appendix 3**. Additional analyses

**Respondent Characteristics**

**Appendix Table 1.** Gender

|  | **Sample** (n = 992) | **GMC Registry** (n = 70,403) |
| --- | --- | --- |
| **Woman** | 486 (48%) | 40,725 (58%) |
| **Man** | 506 (50%) | 29,678 (42%) |

**Appendix Table 2.** Age range

|  | **Sample** (n = 1,005) | **GMC Registry** (n = 70,403) |
| --- | --- | --- |
| **34 years or younger** | 86 (9%) | 7,638 (11%) |
| **35 – 44 years** | 373 (37%) | 26,275 (37%) |
| **45 – 54 years** | 366 (36%) | 20,804 (30%) |
| **55 years or older** | 180 (18%) | 15,686 (22%) |

*Note*: Age ranges in the survey were *‘35 or younger’*, *‘36 – 45’*, *‘56 – 55’*, and *‘56 or older’*.

**Appendix Table 3.** Region

|  | **Sample** (n = 1,005) | **GMC Registry** (n = 70,403) |
| --- | --- | --- |
| **England** | 831 (83%) | 58,801 (83%) |
| North West | 123 (12%) | 7,618 (11%) |
| North East and Yorkshire | 135 (13%) | 8,460 (12%) |
| Midlands | 154 (15%) | 10,386 (15%) |
| East of England | 84 (8%) | 5,982 (8%) |
| London | 115 (11%) | 10,351 (15%) |
| South East | 131 (13%) | 9,319 (13%) |
| South West | 89 (9%) | 6,685 (10%) |
| **Northern Ireland** | 34 (3%) | 2,025 (3%) |
| **Scotland** | 91 (9%) | 6,633 (9%) |
| **Wales** | 49 (5%) | 2,944 (4%) |


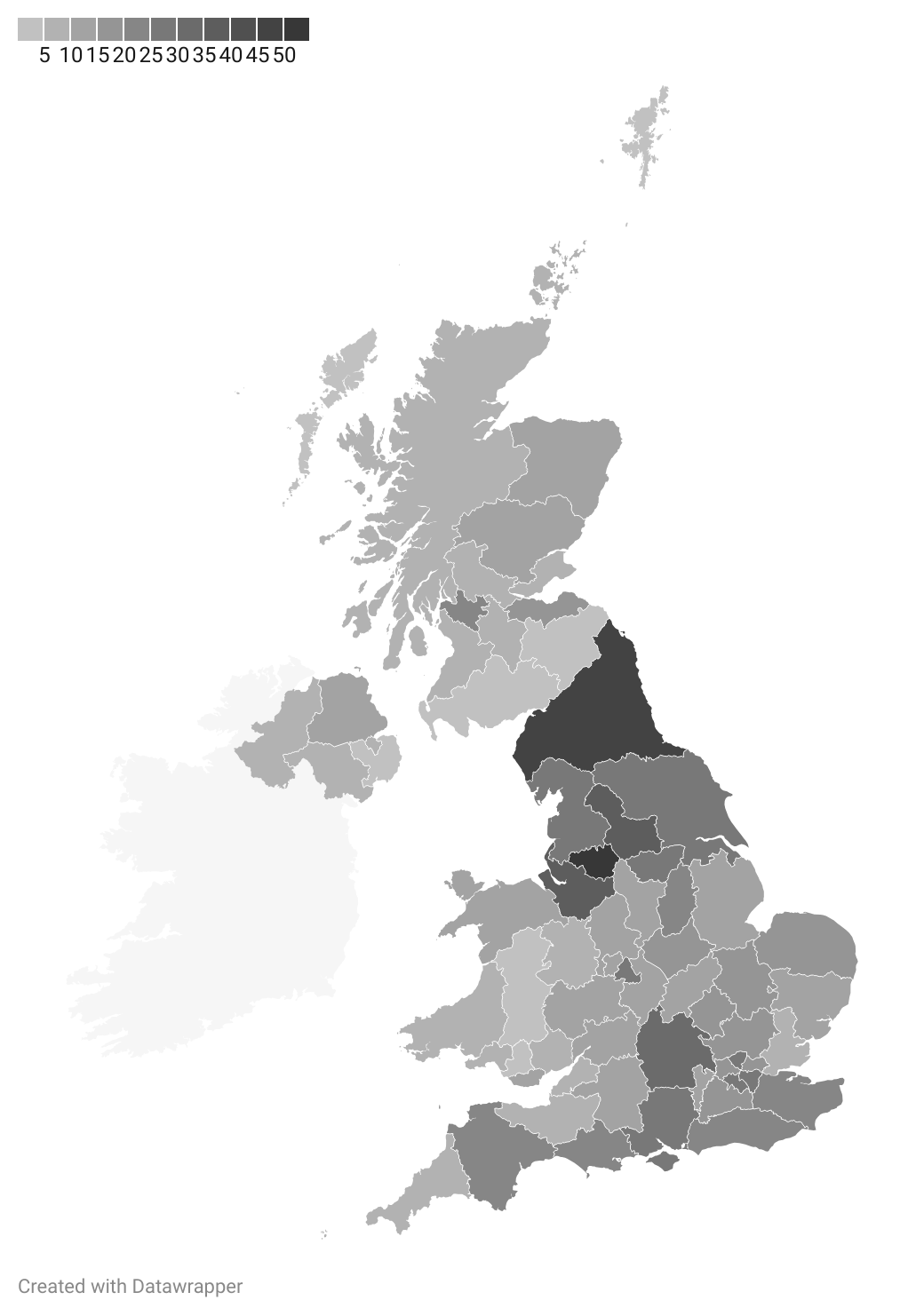


**Figure 1.** Geographical distribution of participated GPs by NHS Integrated care systems, Boards, and Trusts.

**Generative AI in the workplace**

**Appendix Table 4.** Generative AI in the workplace.

|  | Count | % |
| --- | --- | --- |
| **Employer encouraged generative AI tools** |  |  |
| Yes | 107 | 11% |
| No | 850 | 85% |
| Don’t know | 48 | 5% |
| **Employer provided training for AI tools** |  |  |
| Yes | 52 | 5% |
| No | 953 | 95% |
| **Employer prohibited generative AI tools** |  |  |
| Yes | 35 | 3% |
| No | 913 | 91% |
| Don’t know | 57 | 6% |

**GPs’ opinions about generative AI**

**Appendix Table 5.** Opinions about how generative AI tools will improve various aspects of GP work.

|  | **Disagreement** | | | **Agreement** | | | **Don’t know** |
| --- | --- | --- | --- | --- | --- | --- | --- |
|  | Strongly disagree | Disagree | Somewhat disagree | Somewhat agree | Agree | Strongly agree |  |
| **Patient information gathering** | 30 (3%) | 69 (7%) | 117 (12%) | 298 (30%) | 215 (21%) | 60 (6%) | 216 (21%) |
| **Diagnostic accuracy** | 32 (3%) | 102 (10%) | 162 (16%) | 298 (30%) | 114 (11%) | 32 | 265 (26) |
| **Creation of personalised treatment plans** | 50 (5%) | 93 (9%) | 152 (15%) | 303 (30%) | 132 (13%) | 30 (3%) | 245 (24%) |
| **Prognostic accuracy** | 42 (4%) | 86 (9%) | 155 (15%) | 308 (31%) | 109 (11%) | 29 (3%) | 276 (27%) |
| **Conveying empathy** | 326 (32%) | 253 (25%) | 133 (13%) | 79 (8%) | 29 (3%) | 11 (1%) | 174 (17%) |
| **Documentation** | 21 (2%) | 36 (4%) | 69 (7%) | 256 (25%) | 289 (29%) | 149 (15%) | 185 (18%) |
| **Patient communication** | 87 (7%) | 139 (14%) | 147 (15%) | 252 (25%) | 146 (15%) | 34 (3%) | 200 (20%) |
| **Communication with other healthcare providers** | 43 (4%) | 90 (9%) | 128 (13%) | 285 (28%) | 148 (15%) | 50 (5%) | 261 26%) |

**Appendix Table 6.** Opinions about how generative AI tools will impact various aspects of healthcare.

|  | **Disagreement** | | | **Agreement** | | | **Don’t know** |
| --- | --- | --- | --- | --- | --- | --- | --- |
|  | Strongly disagree | Disagree | Somewhat disagree | Somewhat agree | Agree | Strongly agree |  |
| **Increase errors** | 16 (2%) | 86 (9%) | 246 (24%) | 241 (245) | 71 (7%) | 25 (2%) | 320 (32%) |
| **Decrease patient harm** | 42 (45) | 108 (11%) | 214 (21%) | 232 (23%) | 65 (6%) | 10 (1%) | 334 (33%) |
| **Increase patient privacy** | 135 (13%) | 227 (23%) | 243 (24%) | 74 (7%) | 19 (2%) | 5 (.0%) | 302 (30%) |
| **Increase inequities in care delivery** | 28 (3%9 | 78 (8%) | 204 (20%) | 212 (21%9 | 118 (12%) | 41 (4%) | 324 (32%) |
| **Mean more patients will rely on AI tools instead of seeking medical attention** | 20 (25%) | 65 (6%) | 130 (13%) | 337 (34%) | 203 (20%) | 49 (5%) | 201 (20%) |
| **Mean GPs need more support/training in understanding them** | 11 (1%) | 21 (2%) | 50 (5%) | 204 (20%) | 308 (31%) | 286 28%) | 125 (12%) |
| **Increase efficiencies in healthcare** | 25 (2%) | 55 (5%) | 121 (12%) | 363 (365) | 170 (17%) | 58 (6%) | 213 (21%) |

**Appendix Table 7.** Median agreement ratings.

|  | **Median rating** | **IQR [Q1 — Q3]** |
| --- | --- | --- |
| **Improvement of GP work** |  |  |
| Patient information gathering | 4 — Somewhat agree | 2 [3 – 5] |
| Diagnostic accuracy | 4 — Somewhat agree | 1 [3 – 4] |
| Creation of personalised treatment plans | 4 — Somewhat agree | 1 [3 – 4] |
| Prognostic accuracy | 4 — Somewhat agree | 1 [3 – 4] |
| Conveying empathy | 2 — Disagree | 2 [1 – 3] |
| Documentation | 5 — Agree | 1 [4 – 5] |
| Patient communication | 4 — Somewhat agree | 2 [2 – 4] |
| Communication with other healthcare providers | 4 — Somewhat agree | 2 [3 – 5] |
| **Impact on healthcare** |  |  |
| Increase errors | 3 — Somewhat disagree | 1 [3 – 4] |
| Decrease patient harm | 3 — Somewhat disagree | 1 [3 – 4] |
| Increase patient privacy | 2 — Disagree | 1 [2 – 3] |
| Increase inequities in care delivery | 4 — Somewhat agree | 1 [3 – 4] |
| Mean more patients will rely on AI tools instead of seeking medical attention | 4 — Somewhat agree | 2 [3 – 5] |
| Mean GPs need more support/training in understanding them | 5 — Agree | 2 [4 – 6] |
| Increase efficiencies in healthcare | 4 — Somewhat agree | 2 [3 – 5] |

*Note*: *‘Don’t know’* responses were excluded in the calculation of median ratings. The numerical ratings had the following labels: 1 – *‘Strongly disagree’*, 2 – *‘Disagree’*, 3 – *‘Somewhat disagree’*, 4 – *‘Somewhat agree’*, 5 – *‘Agree’*, 6 – *‘Strongly agree’*.

**Appendix Table 8.** Proportion of all agreement responses in 2024 and 2025 surveys.

|  | **2024** (*n* = 1,006) | **2025** (*n* = 1,005) | **Trend** |
| --- | --- | --- | --- |
| **Improvement of GP work** |  |  |  |
| Patient information gathering | 558 (56%) | 573 (57%) | **≈** |
| Diagnostic accuracy | 400 (40%) | 444 (44%) | **↗** |
| Creation of personalised treatment plans | 408 (41%) | 465 (46%) | **↗** |
| Prognostic accuracy | 381 (38%) | 446 (44%) | **↗** |
| Conveying empathy | 149 (15%) | 119 (12%) | **≈** |
| Documentation | 592 (59%) | 694 (69%) | **↗** |
| Patient communication | — | 432 (43%) | — |
| Communication with other healthcare providers | — | 483 (48%) | — |
| **Impact on healthcare** |  |  |  |
| Increase errors | — | 337 (34%) | — |
| Decrease patient harm | — | 307 (31%) | — |
| Increase patient privacy | — | 98 (10%) | — |
| Increase inequities in care delivery | 551 (55%) | 371 (37%) | **↘** |
| Mean more patients will rely on AI tools instead of seeking medical attention | 623 (62%) | 589 (59%) | **≈** |
| Mean GPs need more support/training in understanding them | 806 (80%) | 798 (79%) | **≈** |
| Increase efficiencies in healthcare | 472 (47%) | 591 (59%) | **↗** |

*Note:* Agreement responses were 4 – *‘Somewhat agree’*, 5 – *‘Agree’*, and 6 – *‘Strongly agree’*. Increase (**↗**) or decrease (**↘**) in agreement was defined as more than 3% difference, and difference equal to or less than 3% was noted as comparable (**≈**).
